# ﻿A new species of the genus *Ivela* Swinhoe (Lepidoptera, Erebidae, Lymantriinae) from Guangdong, China

**DOI:** 10.3897/zookeys.1097.79109

**Published:** 2022-04-20

**Authors:** Lin-Zhe Xie, Kun-Yuan Li, Liu-Sheng Chen, Hou-Shuai Wang

**Affiliations:** 1 Department of Entomology, College of Plant Protection, South China Agricultural University, Guangzhou 510642, China South China Agricultural University Guangzhou China; 2 Guangdong Academy of Forestry, Guangzhou 510642, China Guangdong Academy of Forestry Guangzhou China

**Keywords:** adults, *Dendrophleps*, Leucomini, molecular data, phylogenetic analyses, pupae

## Abstract

*Ivelayini***sp. nov.**, is described from Guangdong, China based on morphological characters and molecular data. Adults, including genitalia and wing venation, and pupa are illustrated and compared to those of similar species. A key to Chinese *Ivela* species is provided. Assignment of the new species to *Ivela* Swinhoe is based primarily on a molecular phylogenetic analysis and is corroborated by morphology. Life histories of *I.yini* and *Dendrophlepssemihyalina* Hampson are discussed.

## ﻿Introduction

The tribe Leucomini of Lymantriinae (Erebidae), proposed by Hollоway (1999), is mainly distributed in the Oriental tropics and contains approximately 60 species in four genera (Wang et al. 2015). Adults of this tribe can be recognized by their appearance, often pale white, with translucent areas in male wings, and asymmetric male genitalia (Holloway 1999). Prior to this study, *Ivela* Swinhoe contained three species: *I.auripes* (Butler), *I.ochropoda* (Eversmann), and *I.eleuterioides* (Semper). Of these, *I.auripes* and *I.ochropoda* occur in China.

We report the discovery of a previously unrecognized species of tussock moth that resembles *Dendrophlepssemihyalina* Hampson. Based on multiple morphological characters of adults and pupae and molecular data of four gene regions, we describe it as a species of *Ivela*.

## ﻿Materials and methods

### ﻿Collecting and morphology

All examined specimens were collected in light traps. They are deposited in the Insect Collection of Department of Entomology, South China Agricultural University (**SCAU**), Guangzhou, China. Adults and genitalia were treated following Wang et al. (2010, 2014). Terminology follows Holloway (1999) and Chao (2003).

### ﻿Molecular taxa sampling

We sampled six species, including the type species of all genera of Leucomini, with two species of *Lymantria* Hübner as outgroups. Most sequences of Leucomini and those of the outgroup taxa were downloaded from NCBI. The detailed sampling data for molecular analyses are provided in Table 1.

### ﻿Molecular data analyses

DNA was extracted from two or three legs of adult specimens using the TIANGEN DNA extraction kit following the manufacturer’s instructions. One mitochondrial gene, DNA barcode region of cytochrome c oxidase subunit I (COI), and three nuclear genes, Elongation factor-1 alpha (EF-1α), ribosomal protein S5 (RpS5), and wingless (WNT), were amplificated and sequenced following Folmer et al. (1994) and Wahlberg and Wheat (2008). Concatenation and sequence alignment was performed using MEGA X (Kumar et al. 2018).

A neighbor-joining (NJ) analysis of DNA barcode was performed with MEGA X under the Kimura 2-Parameter (K2P) model (Kimura 1980), and bootstrap values were calculated with 1,000 replicates. A maximum-likelihood (ML) analysis was performed using IQ-TREE (Nguyen et al. 2015) with 1,000 bootstrap replicates, and the best-fitting model was automatically selected by ModelFinder (Kalyaanamoorthy et al. 2017) implemented in IQ-TREE. A Bayesian-inference (BI) analysis was performed using MrBayes 3.2.6 (Ronquist et al. 2012) under the GTR + F + G4 model, with two parallel runs for 2,000,000 generations. The first 25% of trees were discarded as burn-in, and the remaining trees were used to calculate posterior probabilities (PP).

**Table 1.** Sampling data used for molecular analyses in this study.

Specimen voucher no.	Taxa	Locality	GenBank accession no.			
			COI	EF1-a	RPS5	WNT
LE114	*Ivelayini* **sp. nov.**	Guangdong, China	OM242956 ^#^	‒	‒	‒
LE074	*Ivelayini* **sp. nov.**	Guangdong, China	OM242952 ^#^	‒	‒	‒
LE118	*Ivelayini* **sp. nov.**	Guangdong, China	OM242955 ^#^	‒	‒	‒
H340	*Ivelayini* **sp. nov.**	China	KP081829.1	KP082270.1	‒	KP082761.1
LE124	Ivelaauripes*	Guangdong, China	OM242951 ^#^	‒	‒	‒
H49	Ivelaauripes*	China	KP081830.1	KP082302.1	‒	KP082762.1
H181	Perinanuda*	Guangdong, China	KP081831.1	KP082248.1	KP082623.1	KP082763
LE014	Dendrophlepssemihyalina*	Guangdong, China	OM250083 ^#^	OM328195 ^#^	OM328197 ^#^	OM328196 ^#^
LE115	Dendrophlepssemihyalina*	Guangdong, China	OM242954 ^#^	‒	‒	‒
LE116	Dendrophlepssemihyalina*	Guangdong, China	OM242953 ^#^	‒	‒	‒
GD385	Dendrophlepssemihyalina*	Guangdong, China	OM242949 ^#^	‒	‒	‒
H377	*Leucoma* sp.	China	KP081825.1	KP082289.1	KP082620.1	KP082757.1
H351	Leucomasalicis*	China	KP081826.1	KP082276.1	KP082621.1	KP082758.1
H127	*Lymantriadissoluta*	China	KP081854.1	KP082225	KP082643.1	KP082781
H58	*Lymantriasimilis*	China	KP081855.1	KP082304.1	KP082644.1	KP082782.1

* Type species of genus. # Sequences obtained in this study. ‒ No data available.

## ﻿Results

### ﻿Phylogenetic relationships

**Figure 1.**
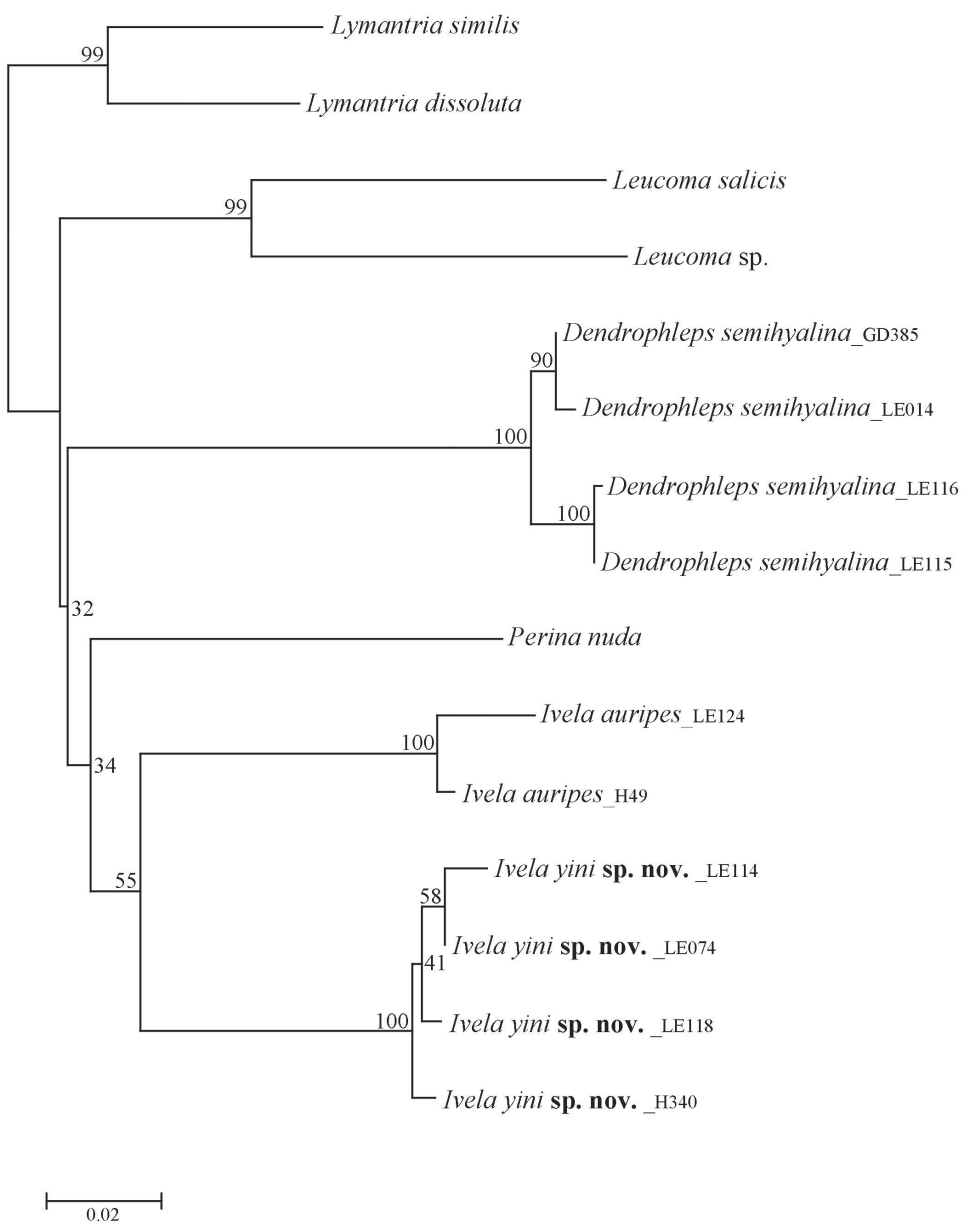
NJ tree of the selected samples of Leucomini based on DNA barcode data. Numbers near nodes represent support values.

**Figure 2.**
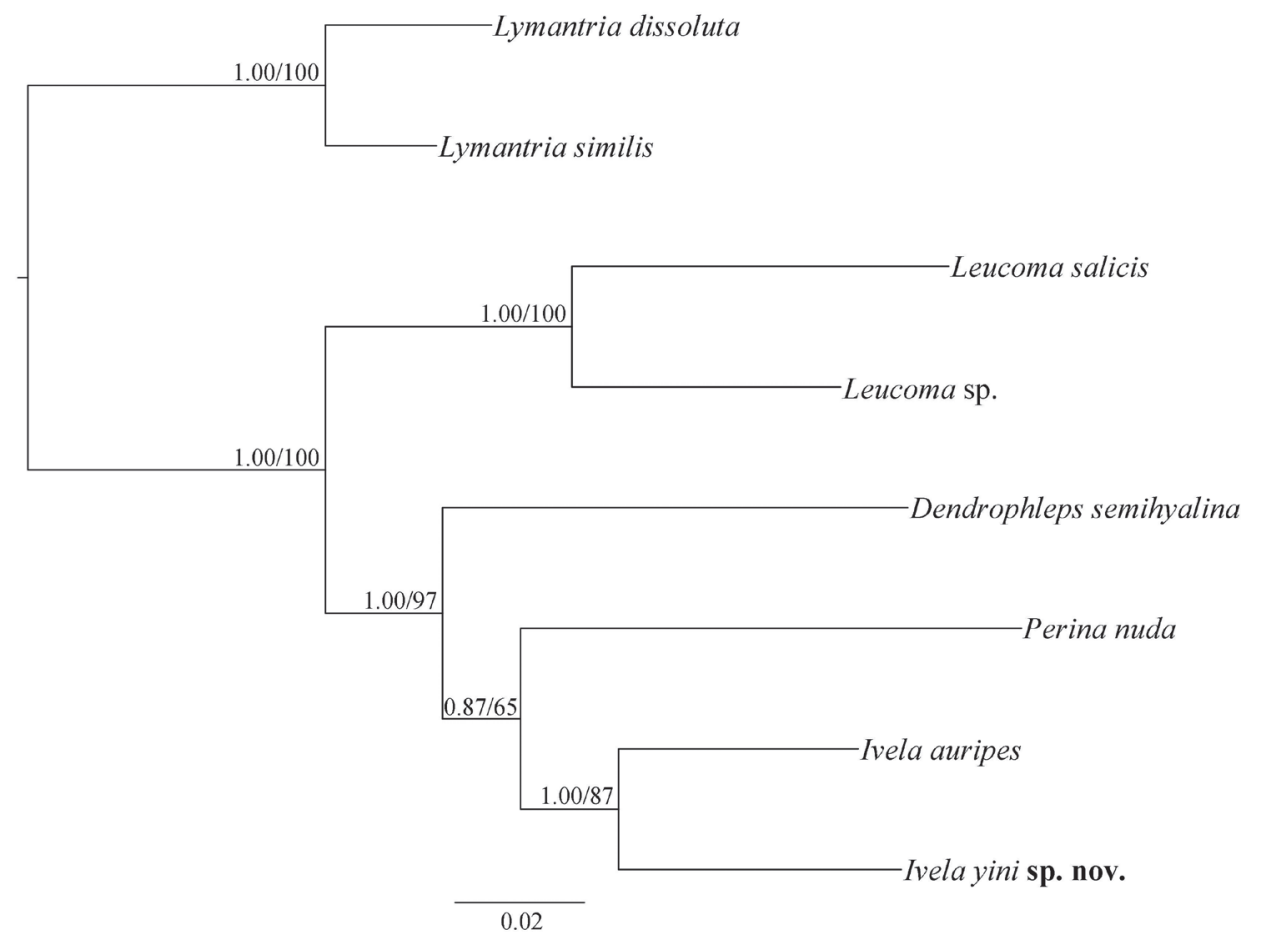
BI tree of the selected species of Leucomini inferred from the combined COI, EF-1α, RPS5, and WNT genes. Posterior probabilities from BI analysis and bootstrap values from ML analysis are indicated above the branches as PP/BP.

The genetic distances of the DNA barcode data (a 658 bp region of the COI gene) of Leucomini species in China are given in Appendix 1. The interspecific genetic distances within *Ivela* ranged from 10.6 to 12.2% (*I.yini* sp. nov. and *I.auripes*); the intraspecific genetic distances from 1.1% (*I.yini*) to 1.9% (*D.semihyalina*); and the intergeneric genetic distances within Leucomini ranged from 12.0% (*I.yini* and *Perinanuda* (Fabricius)) to 19.3% (*Leucomasalicis* (Linnaeus) and *D.semihyalina*). The concatenated dataset of four genes consists of 2,851 nucleotide positions (658 bp for COI, 400 bp for WNT, 600 bp for RPS5 and 1,193 bp for EF-1α). The NJ analysis of the DNA barcode data indicates that the new species and *I.auripes* (the type species of *Ivela*) form a clade in Leucomini (Fig. 1). This clade is strongly supported by both BI and ML analyses of the concatenated dataset (Fig. 2: BP = 1.00, PP = 87).

### ﻿A key to *Ivela* from China

**Table:** 

1	Forewings with R_3_ and R_4_ coincident	***I.auripes***
–	Forewings with R_3_ and R_4_ separated at near apex	**2**
2	Palpi white	***I.yini***
–	Palpi yellow	***I.ochropoda***

### ﻿Species accounts

#### ﻿ Ivela yini

Taxon classificationAnimaliaLepidopteraErebidae

Xie & Wang sp. nov.

A836E550-60C3-526A-BB28-F0D1FACC1A74

http://zoobank.org/2BA5C644-7CCA-4686-A445-6395DFB1E239

Figs 3–6 , 9–11 , 14 , 15–16 , 21–24

##### Diagnosis.

**Figures 3–8.**
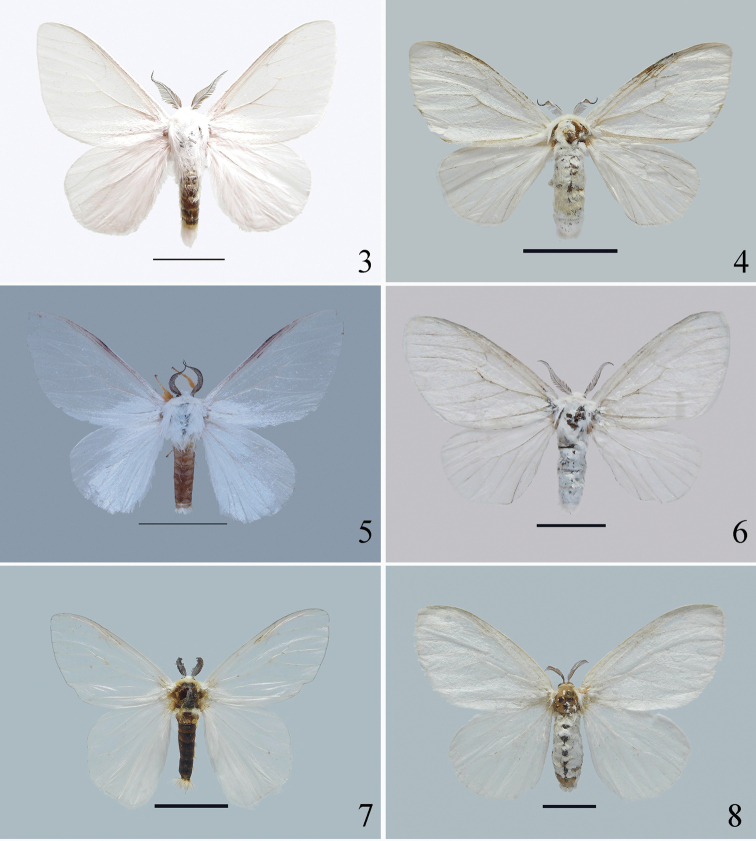
Adults **3‒6***Ivelayini* sp. nov. (**3** male, holotype **4** female, paratype **5** male, paratype **6** female, paratype) **7, 8***Dendrophlepssemihyalina* (**7** male **8** female). Scale bars: 10 mm.

**Figures 9–13.**
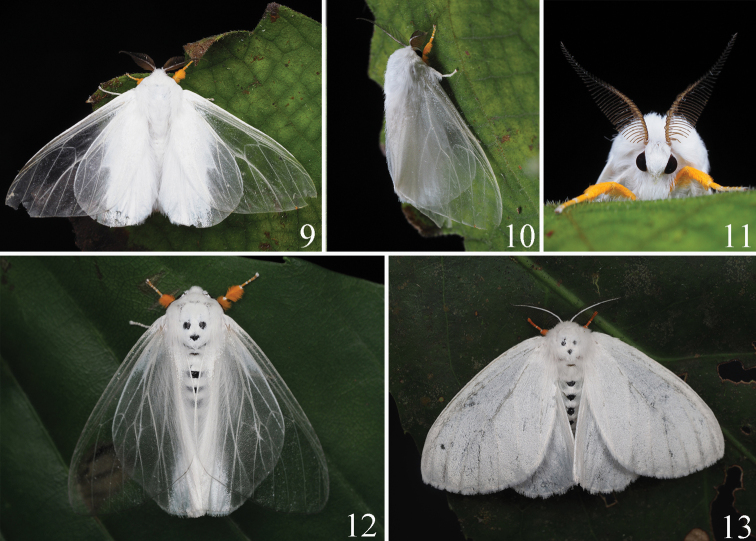
Field images of adults **9‒11***Ivelayini* sp. nov. male (**9** dorsal view **10** lateral view **11** ventral view of head) **12, 13***Dendrophlepssemihyalina* (**12** male, dorsal view **13** female, dorsal view).

This new species is diagnosed by a combination of characters. Superficially, the thorax and abdomen of the adult are white without black markings (Figs 3–6, 9, 10), the palpi are white (Fig. 11), and the forelegs are orange with white rings on the tarsal segments (Figs 9–11). In the male, the asymmetrical valvae are wide and truncated, with a deeply concave cucullus (Fig. 15). The uncus of *I.yini* (Fig. 15) is more than twice as long as the uncus of *I.auripes* (Fig. 17) and *I.ochropoda* (Inoue 1956: fig. 25). The female corpus bursae of *I.yini* has a pair of caudal projections (Fig. 16). The pupa has white hairs on the prothorax, on segments A2 and A3, and near the posterior end (Figs 21–24).

*Dendrophlepssemihyalina* has black markings on the thorax and abdomen (Figs 7, 8, 12, 13), and its valvae are long, narrow, and without a deeply concave cucullus (Fig. 19). The corpora bursae of *I.auripes* and *D.semihyalina* lack caudal projections (Figs 18, 20).

##### Description.

**Adult** (Figs 3–6, 9–11, 14–16).

***Head*** (Fig. 11). Antennae bipectinate, fuscous; frons and vertex covered densely with white hairs; labial palpi white, short.

**Figure 14.**
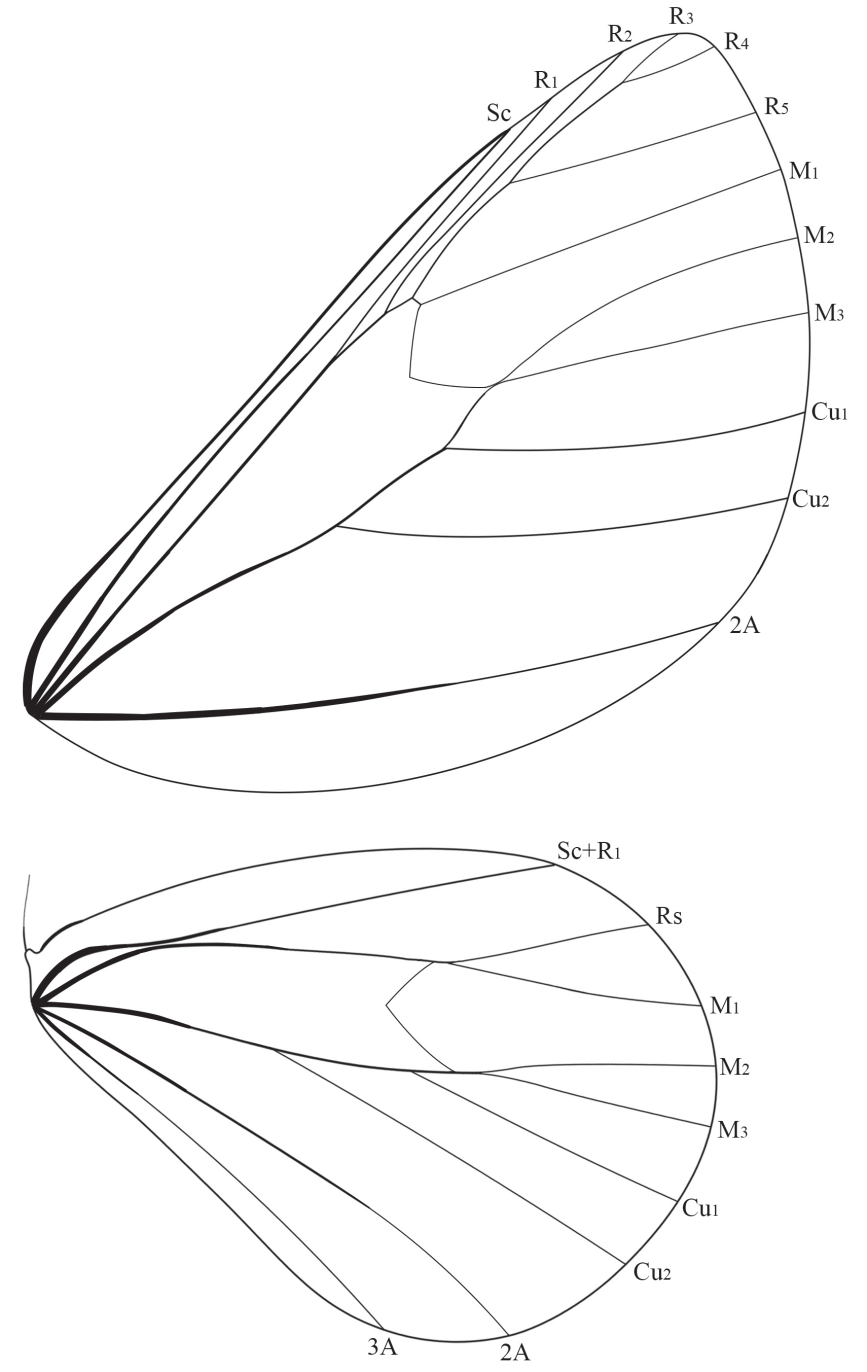
Wing venation of *Ivelayini* sp. nov. (male, paratype).

***Thorax*** (Figs 3–6, 9, 10, 14). Dorsum and venter covered with white scales, tegula white. Forewing length: 39‒42 mm male, 48‒50 mm female. Forewings translucent with dense white scales at basal area in male, white in female; R_1_ and R_2_ almost parallel, R_3,_ R_4_, and R_5_ stalked, M_1_ arising from upper angle of discal cell, M_2_ and M_3_ arising from the lower angle of discal cell respectively, Cu_1_ and Cu_2_ approximately parallel, fringe white. Hindwings white, with a transparent area near apex in male; Rs and M_1_ short stalked, M_2_ and M_3_ short stalked in male but arising separately from the lower angle of discal cell in female, fringe white. Forelegs densely covered with orange scales, tarsi with white rings; mid- and hindlegs white, tarsi yellow with white rings but inconspicuous in male.

**Figures 15–20.**
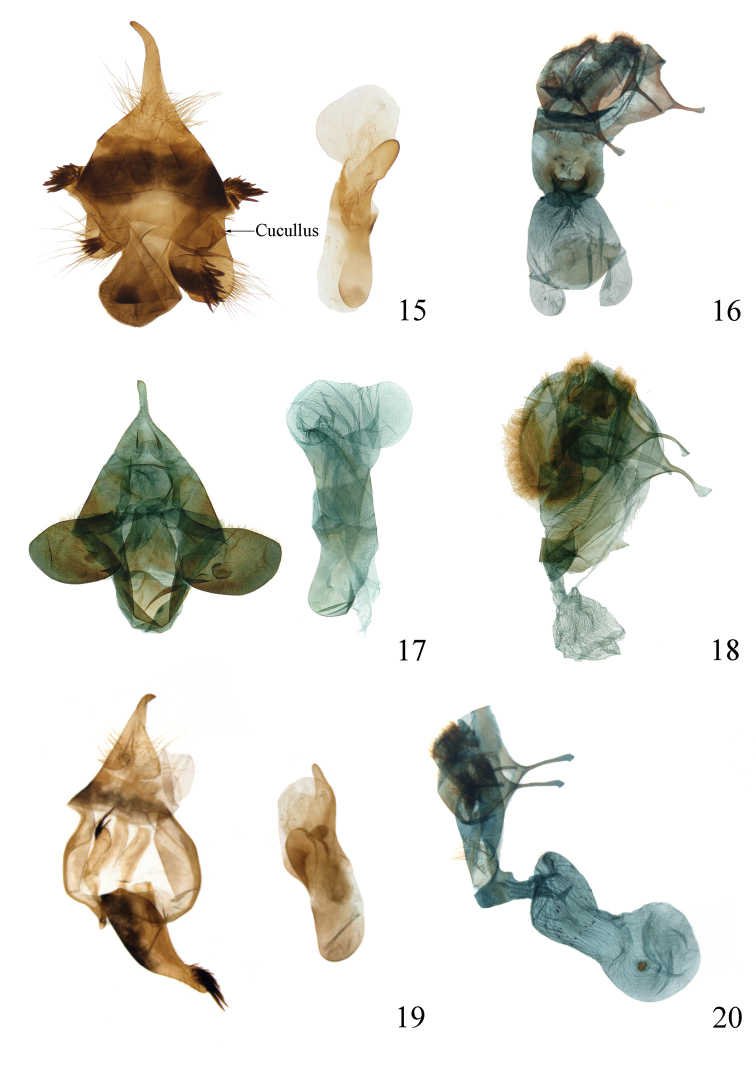
Genitalia **15, 16***Ivelayini* sp. nov. (**15** male, holotype **16** female, paratype) **17, 18***I.auripes* (**17** male **18** female) **19, 20***Dendrophlepssemihyalina* (**19** male **20** female).

***Abdomen. Male genitalia*** (Fig. 15). Uncus hook-shaped apically; tegumen broad; valvae moderately symmetric, left valva smaller than right, broad, extremely short, cucullus concave medially, densely covered with setae on the dorsal and ventral parts of cucullus; saccus well developed; aedeagus tubular, distal gradually slightly curve toward distal area; vesica simple, without cornuti.

***Female genitalia*** (Fig. 16). Anterior apophysis almost as long as posterior apophysis; anal papillae larger; ostium larger; ductus bursae short, sclerotized; corpus bursae with a pair of terminal projections.

**Figures 21–24.**
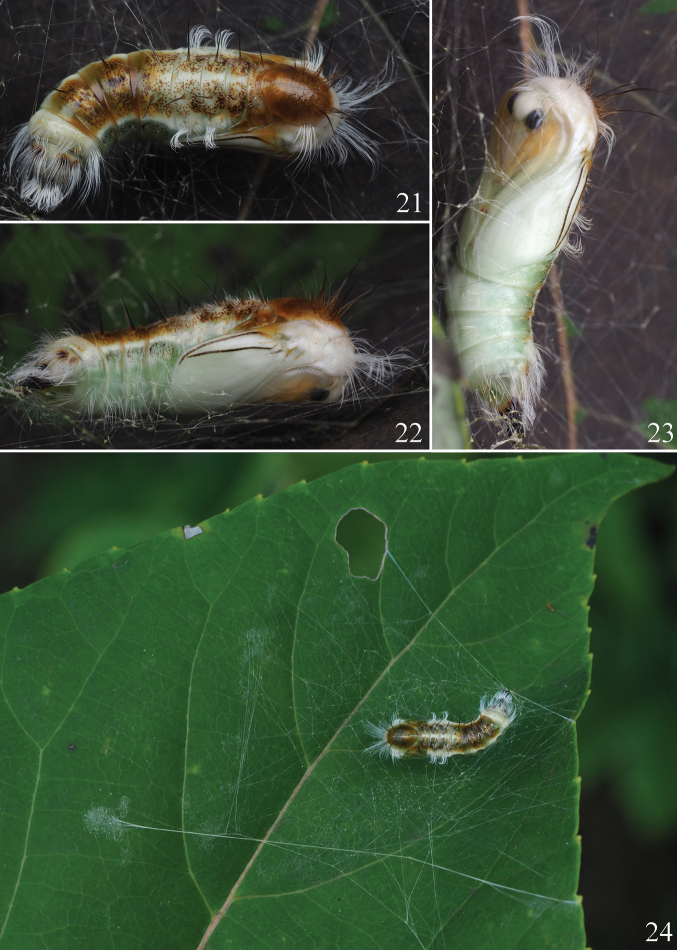
Pupa of *Ivelayini* sp. nov. **21** dorsal view **22** lateral view **23** ventral view **24** pupa on *Idesiapolycarpa* Maxim.

**Pupa** (Figs 21–24). Head white; prothorax white, with long, white hairs; mesothorax and metathorax chestnut-colored on dorsal surface, with dark brown hairs; wings white, with two black lines dorsally; forelegs and midlegs yellow, hindlegs white, with dark yellow dot terminally. Abdomen pale green laterally and ventrally, with chestnut-colored dots and spots dorsally; segments A1–A6 with a pair of black setae; segments A2 and A3 and terminal of abdomen with white hairs.

A single pupa of *I.yini* was discovered on *Idesiapolycarpa* Maxim. (Salicaceae) (Fig. 24). This suggests that this is the foodplant of this species.

##### Habitat.

Forest zone 1000‒1315 m elevation.

##### Materials examined.

***Holotype***: ♂, Nanling National Nature Reserve, Ruyuan County, Guangdong, 25.VI.2008, leg. Min Wang. ***Paratypes***: 1♂, same data as holotype, altitude 1315 m, 12.VII.2010, leg. Min Wang. 1♀, same data as holotype, 3.VII.2011, leg. Min Wang. 1♂, same data as holotype, altitude 1000 m, 10.VII.2019, leg. Ran Yin & Xiao-juan Xing. 1♀, same data as holotype, 11‒14.VI.2019, leg. Hou-shuai Wang.

##### Distribution.

China (Guangdong).

##### Etymology.

The species is named after Ran Yin, who discovered the pupa of the new species. The name is in the genitive case.

##### Remarks.

The female genitalia of *I.auripes* (Fig. 18) and *D.semihyalina* (Fig. 20) have to our knowledge not been illustrated previously. They are illustrated here for comparative purposes.

**Figures 25–30.**
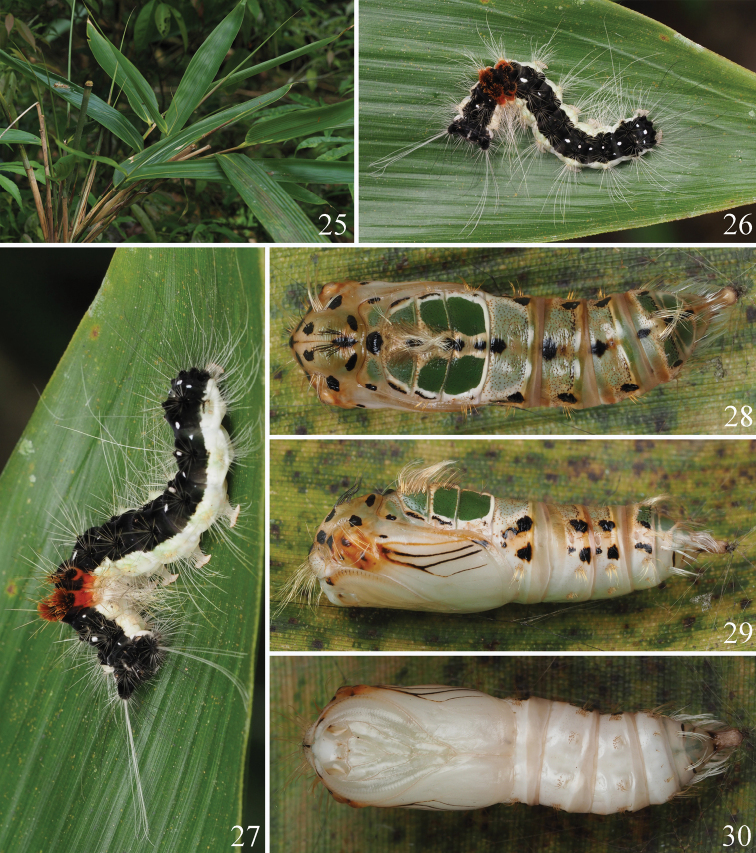
Immature stages and host plant of *Dendrophlepssemihyalina***25** host plant: *Indocalamustessellatus* (Munro) Keng f. **26, 27** last instar larva on the host plant **28‒30** pupa (**28** dorsal view **29** lateral view **30** ventral view).

The early stages of *D.semihyalina* are also newly reported as below (Figs 25–30):

**Host plant of *D.semihyalina*** (Fig. 25): *Indocalamustessellatus* (Munro) Keng f. (Poaceae).

**Last instar larva of *D.semihyalina*** (Figs 26, 27): body white laterally and ventrally; dorsally black, with scattered white dots. A1 and A2 with reddish orange tufts dorsally. Verrucae pale yellow laterally, black dorsally, with long white or black hairs.

**Pupa of *D.semihyalina*** (Figs 28–30): body white laterally and ventrally, green-brown dorsally. Thorax and abdomen with irregular black spots on lateral and ventral surfaces. A1–A3 with a pair of green patches on dorsal surface. Wings with some mixed orange and black veins.

## ﻿Discussion

*Ivelayini* is superficially similar to several tussock moths with which it is sympatric. We illustrated it with *Dendrophlepssemihyalina* and *Ivelaauripes* for comparative purposes. These species can be distinguished reliably by the combinations of superficial characters outlined above. The identification can be confirmed by dissection of the male and female genitalia if required.

The genetic distance values of DNA barcode data between Lepidoptera species are ordinarily greater than 3% (Hebert et al. 2003). Our analysis of Chinese Leucomini indicates that the DNA barcode of *I.yini* is 10.6% from the closest species *I.auripes* (Appendix 1). The NJ tree also strongly supports the validity of the new species (Fig. 1). Our phylogenetic analyses show that *I.yini* and *I.auripes* are a monophyletic clade (PP = 1.00, BP = 87) and strongly suggest that *I.yini* belongs in *Ivela* (Fig. 2). This arrangement is supported by morphology. All *Ivela*, including *I.yini*, share wide valvae, and their hindwings lack a row of oblique veinlets (accessory veins) between A2 and the dorsal margin. These veinlets are considered diagnostic for *Dendrophleps* (Holloway 1999; Mackey 2019).

Several hardwoods were reported as foodplants for *I.auripes: Corylopsis multiflora* Hance (Hamamelidaceae), *Cornuscontroversa* Hemsley (Cornaceae), *C.brachypoda* C.A. Mey (Cornaceae), *Styraxjaponicus* Sieb. et Zucc (Styracaceae), and *S.obassis* Siebold et Zucc (Styracaceae) (Inoue 1956; Chao 2003). While our discovery of a pupa of *I.yini* on *Idesiapolycarpa* is less than absolute proof that it is the foodplant of this moth, it does suggest that *I.yini* feeds on a broadleaved tree. In contrast, *D.semihyalina* was discovered to be a grass feeder. These foodplant differences support placement of these similar-appearing moths into different genera.

## Supplementary Material

XML Treatment for Ivela yini

## ﻿Appendix 1

**Table A1.** Kimura 2-parameter genetic distances based on COI barcodes among 13 samples of Leucomini and two outgroups.

Species code	Species name	1	2	3	4	5	6	7	8	9	10	11	12	13	14
LE114	*Ivelayini* **sp. nov.**														
LE074	*Ivelayini* **sp. nov.**	0.006													
LE118	*Ivelayini* **sp. nov.**	0.000	0.006												
H340	*Ivelayini* **sp. nov.**	0.011	0.011	0.011											
LE124	*Ivelaauripes*	0.122	0.119	0.122	0.120										
H49	*Ivelaauripes*	0.110	0.106	0.110	0.108	0.020									
H181	*Perinanuda*	0.126	0.122	0.126	0.120	0.157	0.144								
LE116	*Dendrophlepssemihyalina*	0.154	0.156	0.154	0.154	0.183	0.165	0.170							
LE115	*Dendrophlepssemihyalina*	0.152	0.154	0.152	0.152	0.181	0.163	0.168	0.002						
GD385	*Dendrophlepssemihyalina*	0.144	0.146	0.144	0.144	0.167	0.150	0.161	0.019	0.017					
LE014	*Dendrophlepssemihyalina*	0.142	0.144	0.142	0.142	0.165	0.148	0.159	0.015	0.014	0.003				
H377	*Leucoma* sp.	0.169	0.173	0.169	0.171	0.174	0.155	0.172	0.187	0.187	0.182	0.181			
H351	*Leucomasalicis*	0.159	0.161	0.159	0.161	0.163	0.149	0.182	0.193	0.191	0.188	0.187	0.127		
H127	*Lymantriadissoluta*	0.138	0.135	0.138	0.142	0.153	0.136	0.131	0.157	0.155	0.149	0.147	0.159	0.167	
H58	*Lymantriasimilis*	0.133	0.129	0.133	0.133	0.139	0.121	0.137	0.154	0.154	0.146	0.145	0.144	0.163	0.071
